# Neighborhood Prices of Healthier and Unhealthier Foods and Associations with Diet Quality: Evidence from the Multi-Ethnic Study of Atherosclerosis

**DOI:** 10.3390/ijerph14111394

**Published:** 2017-11-16

**Authors:** David M. Kern, Amy H. Auchnicloss, Mark F. Stehr, Ana V. Diez Roux, Latetia V. Moore, Genevieve P. Kanter, Lucy F. Robinson

**Affiliations:** 1 Department of Epidemiology and Biostatistics, Dornsife School of Public Health, Drexel University, Philadelphia, PA 19140, USA; dmk42@drexel.edu (D.M.K.); lfr32@drexel.edu (L.F.R.); 2 School of Economics, LeBow College of Business, Drexel University, Philadelphia, PA 19104, USA; ms424@drexel.edu; 3 Dean’s Office, Epidemiology and Biostatistics, Urban Health Collaborative, Dornsife School of Public Health, Drexel University, Philadelphia, PA 19104, USA; avd37@drexel.edu; 4 Division of Nutrition, Physical Activity & Obesity Prevention & Control, National Center for Chronic Disease Prevention & Health Promotion, Centers for Disease Control & Prevention, Atlanta, GA 30329, USA; lvmoore@cdc.gov; 5 Department of Health Management and Policy, Dornsife School of Public Health, Drexel University, Nesbitt Hall, 3215 Market St. Philadelphia, PA 19104, USA; gpkanter@drexel.edu

**Keywords:** diet, food environment, food prices, instrumental variable analysis, nutrition

## Abstract

It is known that the price of food influences the purchasing and consumption decisions of individuals; however, little work has examined if the price of healthier food relative to unhealthier food in an individual’s neighborhood is associated with overall dietary quality while using data from multiple regions in the United States. Cross-sectional person-level data came from The Multi-Ethnic Study of Atherosclerosis (exam 5, 2010–2012, *n* = 2765); a food frequency questionnaire assessed diet. Supermarket food/beverage prices came from Information Resources Inc. (*n* = 794 supermarkets). For each individual, the average price of select indicators of healthier foods (vegetables, fruits, dairy) and unhealthier foods (soda, sweets, salty snacks), as well as their ratio, was computed for supermarkets within three miles of the person’s residential address. Logistic regression estimated odds ratios of a high-quality diet (top quintile of Healthy Eating Index 2010) associated with healthy-to-unhealthy price ratio, adjusted for individual and neighborhood characteristics. Sensitivity analyses used an instrumental variable (IV) approach. Healthier foods cost nearly twice as much as unhealthier foods per serving on average (mean healthy-to-unhealthy ratio = 1.97 [SD 0.14]). A larger healthy-to-unhealthy price ratio was associated with lower odds of a high-quality diet (OR = 0.76 per SD increase in the ratio, 95% CI = [0.64–0.9]). IV analyses largely confirmed these findings although—as expected with IV adjustment—confidence intervals were wide (OR = 0.82 [0.57–1.19]). Policies to address the large price differences between healthier and unhealthy foods may help improve diet quality in the United States.

## 1. Introduction

An individual’s healthy food environment encompasses more than just physical access to fruits, vegetables, and other nutritious foods. Economic access and the affordability of healthy foods in the neighborhood is also a key component of one’s food environment. The price of food—in addition to taste, nutrition, convenience, and other factors—affects the purchasing choices of individuals who must navigate environments with numerous choices and saturated by advertising [1,2,3,4]. Healthier foods have been found to be more expensive than less healthy foods, when measured per calorie [5,6] or per serving [7,8,9,10] but not by weight [7]. For this reason, low cost diets are associated with higher calorie intake at the expense of fewer nutrients [6], while healthier diets tend to be more expensive [11,12,13,14]. Additionally, the price of food has been found to be associated with blood cholesterol levels [15], blood glucose levels [16], and obesity [17]. While prior work has shown that higher quality diets cost more than poorer quality diets, there is little evidence examining the association between local food prices across multiple regions and overall diet quality. 

A healthy diet, as measured by the Healthy Eating Index (HEI, an energy-adjusted score), is inversely associated with obesity [18], waist circumference [19], diabetes [20], cardiovascular disease [20,21], stroke [20], cancer [22,23], and mortality [24,25,26,27]. The average consumer’s diet falls well short of the U.S. Department of Agriculture and U.S. Department of Health and Human Services recommendations [28]. This may be due in part to purchasing too few fresh fruits and vegetables, and instead, purchasing more affordable highly processed fats, sugars, and sweets.

Lower socioeconomic status (SES) [29,30,31] has been associated with decreased consumption of fruits and vegetables, and prior work has found that these SES-differences can be explained primarily by the cost of a diet [32,33]. It is reasonable to expect that large differences in price between healthy and unhealthy foods would lead to differences in purchasing patterns and resulting diets, and that those differences would be more prominent for individuals of lower SES [17,34]. An experimental study found that food taxation and subsidy policies have a differential effect by income level [35], while an observational study found that the association between metropolitan area food prices and diet quality was not modified by income level [36]. However, there is a lack of research examining how local neighborhood food prices may affect diet quality differently across levels of SES.

This cross-sectional study spatially linked 2765 participants from the Multi-Ethnic Study of Atherosclerosis (MESA) to nearby supermarkets to examine the association between neighborhood food price and diet quality. 

## 2. Materials and Methods

### 2.1. Subjects (MESA Data)

MESA is a population-based longitudinal cohort study of ethnically diverse adults aged 45–84 years [37] with no known presence of cardiovascular disease. Individuals were recruited from six sites across the United States: Bronx/Upper Manhattan, NY; Baltimore City and Baltimore County, Maryland; Forsyth County, North Carolina; Chicago, Illinois; St. Paul, Minnesota; and Los Angeles County, California. MESA included a baseline examination (2000–2002) and four follow-up exams; 4716 respondents participated in exam 5 (2010–2012). Written informed consent was obtained from the participants, and the study was approved by institutional review boards at each site (Drexel University IRB Number: 11404002814 (Social, biologic and geographic factors in cardiovascular disease)). Ultimately, nearly all individuals from St. Paul were not included in the analysis because supermarket data was not available in that area. The handful of individuals from the St. Paul site who were included were those who had moved out of the St. Paul metropolitan area and to a neighborhood for which supermarket data was available. Individuals included in the analysis came from 954 unique census tracts (mean number of individuals per tract = 2.9).

Neighborhood food pricing was available concurrent to exam 5, thus, this study only included participants who completed exam 5, administered during April 2010 through January 2012. MESA person-level data included in this study were: diet (see details below), age, sex, race/ethnicity, smoking status, marital status, body mass index (BMI), physical activity (metabolic equivalent (MET) minutes/week), education level, and income/wealth index (combination of income level and ownership of four assets: car, home, land, and investments) (see Table 1). 

### 2.2. Food Frequency Questionnaire (FFQ) 

Diet was assessed via FFQ: a modified Block-style, 128 item questionnaire. Participants were asked about their usual eating habits over the past 12 months. For each of the food items on the FFQ, respondents chose their consumption frequency (rare or never, 1 per month, 2–3 per month, 1 per week, 2 per week, 3–4 per week, 5–6 per week, 1 per day, and 2+ per day); their frequency of consumption was then weighted by a multiplier, according to their reported typical serving size (×0.5, ×1.0, and ×1.5 for small, medium, and large, respectively). 

The MESA FFQ was adapted from the questionnaire designed for the Insulin Resistance and Atherosclerosis Study (IRAS) [38], and has been described elsewhere [39]. Modifications to the FFQ included additional items to reflect the multi-ethnic composition of the MESA cohort. IRAS was validated against 24 h dietary recalls [38], and the MESA diet data correlated as expected with high-density lipoprotein (HDL) cholesterol and triacylglyceride (TAG) concentrations [40], and cardiometabolic conditions [41,42,43,44,45].

Following work by others, we excluded participants whose dietary data were considered unreliable, due to reporting usual energy intake <600 or >6000 kcal [40].

### 2.3. Outcome: Healthy Eating Index-2010 (HEI)

HEI-2010 was used to assess dietary quality. The HEI reflects 2010 U.S. federal dietary guidelines, has been used to monitor and assess diet quality in the United States [46,47,48], and has (1) adequate content validity [48], (2) sufficient construct validity, and (3) acceptable reliability [49]. The HEI-2010 includes twelve components (whole fruit, total vegetables, sodium, etc.), each of which contribute a minimum of 0 to a maximum of 5, 10, or 20 points, resulting in a range of 0 to 100 for the total score and higher scores indicate a healthier diet [48].

Linkage of MESA food consumption with HEI food composition was done following the protocol established by the National Cancer Institute [48,50]. Each individual’s nutritional values were derived by linking the food items from the FFQ to MyPyramid Equivalents Database (MPED), multiplying by the number of servings reported in the FFQ, and then summing to obtain a value for each component in the HEI, and then calculating the HEI-2010 score.

Preliminary analyses revealed a roughly normal distribution of HEI scores. We ranked the index into quintiles and operationalized “high-quality diet” as the top quintile (>80th percentile; score >69.0) of all scores in the sample. The top quintile has been used to define a healthy diet in prior work [22,26,27], and ranking the dietary values acknowledges the low precision inherent in dietary self-reports [51].

### 2.4. Price Data

Data on neighborhood food prices were obtained from Information Resources Inc. (IRI, Chicago, IL, USA), a market research group that monitors prices of 299 consumer packaged goods sold in large chain supermarkets and superstores across the United States [52,53,54]. Ultimately, data were included from 794 stores (82 chains) located in 11 states (including Washington, DC), 72 counties, and 757 census block groups. Data years were 2009–2012. 

Nine food/beverage product categories were selected to serve as proxies for either healthier or unhealthier foods. Because data for fresh fruits and vegetables were not available, refrigerated products (a proxy for perishable unprepared foods) were selected under the assumption that they had similar spoilage/refrigeration and storage/distribution costs as fresh produce. In general, perishable unprepared foods tend to be healthier. Thus, healthier food was represented by two product classes: (1) dairy (refrigerated milk, yogurt, cottage cheese), and (2) fruits and vegetables (frozen vegetables, fresh orange juice). Orange juice, while not necessarily a healthy food itself, was used as a proxy for fresh oranges, due to the high correlation between the price of fresh oranges and orange juice [55]. Unhealthier food was represented by packaged, highly processed, long-shelf life products: soda, sweets (chocolate candy, cookies), and salty snacks. Products within the healthier and unhealthier domains were weighted according to national consumption averages to create a price per serving index. Further information regarding the price calculations is available in Supplemental Table S1.

Prices in the database did not include taxes and manufacturer coupons, but instead, reflected the shelf price and included store-level promotions. The primary exposure of interest was the price of healthier foods relative to unhealthier foods, which was operationalized as the ratio of the average price per serving of healthier food divided by the average price per serving of unhealthy food. To simplify the terminology, we hereafter use the following terms for food prices: “healthy”, “unhealthy”, and “healthy-to-unhealthy price ratio”. Serving sizes were defined according to the reference amounts customarily consumed according to the FDA [56]. Price per serving was used to facilitate meaningful comparisons across different types of foods [7]. A ratio >1.0 indicates serving of healthy food was more expensive than a serving of unhealthy food. The prices of healthy foods and unhealthy foods were also modeled separately as secondary exposures of interest. Each price exposure was converted to a z-score: a one unit increase in the z-score represented an increase of 14% in the relative price of healthy food compared with unhealthy food for the healthy-to-unhealthy ratio, and an increase of $0.04 and $0.03 in the average price per serving of healthy and unhealthy food prices, respectively.

The average price of brand name toilet paper in stores within three miles of each MESA participant was also calculated, and used as an instrument for unhealthy food price and the price ratio in the sensitivity analyses described in the “Statistical Methods” section.

The number of IRI supermarkets within three miles of each MESA participant’s address at exam 5 was created, and referred to as the supermarket density.

### 2.5. Census Data

U.S. Census data came from the American Community Survey 2007–2011. Geographic regions (Northeast, Midwest, South, West) and population density were assigned to each participant using 2010 Census data. A neighborhood block group SES index was created using six variables representing wealth and income—household income, housing value, investment income, education level, and managerial occupations—and was operationalized as a single continuous measure [57].

### 2.6. Cost of Living Data and Supermarket Density

The cost of living index 2010 was obtained from the Council for Community and Economic Research for each metropolitan area [58].

### 2.7. Data Linkage

Addresses of MESA participants and supermarket addresses from the pricing dataset were used to link individuals to the average food/beverage prices at supermarkets within a three-mile buffer (radius) of each MESA participant residence using ArcGIS 10.0 (Esri, Redlands, CA, USA). Median number of supermarkets per MESA participant in the analytic sample was 5 (25th–75th percentile 3–6). A three-mile radius was used for consistency with other research examining neighborhood food environments, and is in line with prior research estimating the average distance individuals travel to their primary supermarket [59,60,61,62]. Sensitivity analyses using equal weights for each product category within the healthy and unhealthy domains, rather than those based on national consumption averages, using supermarkets within five miles instead of three miles, and using a one-mile buffer for those living in New York City, produced similar results, which can be found in Supplemental Table S3. 

### 2.8. Statistical Methods

Multivariable logistic regression was used to model the relative odds of having a high-quality diet for every standard deviation increase in the healthy-to-unhealthy price ratio. Model 1 adjusted for geographic region, age (continuous), and gender. Model 2 added income/wealth index, education, race/ethnicity, and smoking status, and model 3 added neighborhood level SES and supermarket density. Other variables (marital status and physical activity) were considered as covariates in the model, but were not included, due to a lack of association with the outcome. 

We tested the interaction of price with education level and income/wealth tertile by including appropriate interaction terms (separate models examining education and income/wealth) and conducting stratified analyses by tertile of income/wealth (low, medium, and high) and education level (high school degree or less, some college, or bachelor’s degree or more). Our hypothesis was that the association between price and diet would be stronger for lower levels of SES. 

Sensitivity analyses used an instrumental variable approach to remove potential unmeasured confounders that would affect food prices and diet, such as the local food culture and the types of foods typically available in the neighborhood. The use of instrumental variables (IV) has been increasing in epidemiologic research as a means to estimate causal effects [63,64,65]. Toilet paper price was chosen as the instrument for the price of unhealthy foods, and in turn, the healthy-to-unhealthy price ratio, because it (1) has a strong association with food prices in the same stores (particularly unhealthy long-shelf life foods), (2) has no anticipated causal association with participants’ diet quality, other than through its correlation with food price, and (3) is in principle not associated with unmeasured confounders mentioned above. These characteristics satisfied the three major assumptions of an instrument: (1) it is strongly associated with the exposure, (2) any effect on the outcome is fully mediated by the exposure, and (3) it shares no unmeasured common causes with the outcome [66].

A two-stage residual inclusion (2SRI) analysis was used to perform the instrumental variable analysis. A 2SRI model was used because it is statistically consistent in non-linear models, such as the logit model used in this study, while the two-stage predictor substitution model commonly used in linear models is not [67]. In the first stage, linear regression was used to regress the price ratio on toilet paper price and covariates (age, gender, region, income/wealth index, education, race, smoking status, neighborhood SES, supermarket density, population density, and cost of living). The residuals from the stage 1 regression were then included as a covariate in the second stage. The second stage model used a logit model to regress the indicator for a high-quality diet on the price ratio (the main exposure of interest), the residuals obtained from stage 1, and all covariates used in stage 1. Bootstrapping of 10,000 samples was used to calculate 95% credible intervals for the stage 2 estimates.

An additional sensitivity analysis used hierarchical modeling to account for potential clustering within the same census tract. Results were identical to those without clustering, and thus, are not shown. 

## 3. Results

Among 4716 MESA participants who contributed data to exam 5, 1047 were excluded, due to not having dietary data (see FFQ section in Methods for more details on diet data). Further exclusions were as follows: 835, due to not being within three miles of a supermarket in our dataset (of which 674 were from the St. Paul MESA site where supermarket data were unavailable) and 69 who did not have full covariate data. The final analytic sample was 2765. Sample characteristics for included vs. excluded are shown in Supplemental Table S2. On average, the excluded sample was similar to those who were included on most characteristics. There were differences in regional and racial characteristics, largely due to the exclusion of those living in the St. Paul area, and small differences in education levels, as a smaller proportion of college graduates were in the excluded sample. 

Demographics and other characteristics of the MESA participants are reported in Table 1. Overall, the serving price of healthy food was nearly twice as expensive as unhealthy food (mean ± SD of healthy-to-unhealthy price ratio = 1.97 ± 0.14, and on an absolute scale: $0.60 ± $0.04 vs. $0.31 ± $0.03 per serving for healthy and unhealthy food, respectively, Table 2). While the ratio was analyzed as a continuous z-score in all analytic models, the tertiles of healthy-to-unhealthy price ratio are used in the table for descriptive purposes. Large differences across the tertiles was only apparent for region (highest ratio in west and lowest in south) and for race/ethnicity (Chinese resided in highest ratio areas and Black in lowest ratio areas, Table 1). Prior to adjusting for covariates, the proportion of individuals with a high-quality diet was 24%, for those with the highest healthy-to-unhealthy ratio, compared with 18% of other individuals (Table 2). 

### 3.1. Primary Analysis

Table 3 displays sequential adjustment for covariates in each of the three models. Results of the final model indicate an inverse association between the healthy-to-unhealthy ratio and high-quality diet. That is, for every one standard deviation increase (14% higher) in the price of healthy food to unhealthy food, the odds of having a high-quality diet decreased by 24% (OR = 0.76, 95% CI = 0.64 to 0.91). No association was found between healthy food price alone with diet (OR = 1.04), while a higher price (per standard deviation increase, approximately $0.03) of unhealthy food was associated with increased odds of having a high-quality diet (OR = 1.16, 95% CI = 1.02 to 1.33). 

Healthy and unhealthy prices were highly correlated (Pearson’s r = 0.63) and caution is recommended when interpreting correlated estimates from the same model. Nevertheless, we performed an exploratory analysis, where healthy and unhealthy prices were included in the same model. Results suggested that the price estimates became stronger and the confidence interval became wider, though inference was unchanged. Controlling for healthy food price, the OR for unhealthy food price was 1.35 (95% CI: 1.10 to 1.66); controlling for unhealthy food price, the OR for healthy food price was 0.79 (95% CI: 0.62 to 1.02) (not shown in tables).

There was a statistically significant interaction between individual income/wealth and the price ratio on diet, while adjusting for all covariates included in the final model (*p* for interaction 0.001, Table 4). All stratified point estimates were in the expected direction, but the gradient was somewhat unexpected: the strongest and only statistically significant point estimate was in the middle income/wealth tertile (OR = 0.61). The next largest association was found within the lowest income/wealth tertile (OR = 0.79), while those in the highest tertile had the weakest association (OR = 0.87), as expected. The interaction was not statistically significant between education and the price ratio (*p* for interaction 0.095), nevertheless, stratified estimates showed the weakest association in least educated and the strongest in most educated (OR 0.85 for high school or less, and OR 0.77 for Bachelor’s or more), counter to expectations. 

### 3.2. Instrumental Variable Analyses

Results of the 2SRI IV analysis are shown in Table 5. In stage 1, the instrument, toilet paper price, was positively associated with unhealthy food price (Spearman’s correlation coefficient, r = 0.56) and negatively associated with the healthy-to-unhealthy price ratio (r = −0.41). As expected, the correlation was higher with unhealthy food prices (r = 0.56) than with healthy food prices (r = 0.29). The F-statistic was 1113, and guidelines specify the minimum value should be 10 [68]. In stage 2, the association between healthy-to-unhealthy price ratio and high-quality diet was similar in magnitude to the results obtained in the primary analysis; however, the result was not statistically significant (OR = 0.82, 95% bootstrapped CI = 0.57 to 1.19). 

## 4. Discussion

Higher prices of healthy foods relative to unhealthy foods were found to be associated with lower odds of having a high-quality diet: per 14% higher ratio of healthy food to unhealthy food, there were 24% lower odds of having a high-quality diet. Despite healthy foods costing more per serving than unhealthy foods, there was no association between diet and prices of healthy foods alone, but there was a positive association with unhealthy food price alone (per $0.03 higher unhealthy food price per serving there was a 16% higher odds of having a healthy diet). To our knowledge, no prior study examining healthy and unhealthy food prices has linked neighborhood food prices (rather than aggregate level prices) to a cohort of individuals throughout the United States to examine the association with diet. 

Consumers report that price is a main driver in food purchase decision making [1,51], and our study illustrates how this may impact diet. While much work has been done to improve the quality of diets by understanding and improving the lack of physical access to supermarkets in food deserts [69], additional paths should be considered. Improving the affordability of healthy foods relative to their unhealthy substitutes may be an effective option. Examples of options proposed for improving the relative affordability of healthy foods include taxation of unhealthy foods, subsidizing healthy food, and combinations of these practices. One study suggests taxing unhealthy foods has the benefit of raising revenue, and could be an effective strategy to influence dietary changes [70]. Another study suggests that subsidizing healthy food has the benefit of directly making healthy food more affordable, and appears to increase the consumption of those foods, though with a smaller effect as the unhealthy food taxes [71], and may require government funding. It has been suggested that combining strategies to influence consumption patterns would be much more effective than any single method alone [70,72] and may satisfy consumers generally opposed to “sin” taxes, by providing them simultaneous savings on their grocery bill due to cheaper healthy foods [73].

We expected differences in the price exposure to have a stronger association within those of lower SES; however, our results suggested it was instead those in the middle range of the income/wealth index, and those with the highest level of education for which there was a stronger association. Our results are consistent with findings from a recent study which found food price policies had much larger impacts in middle-income individuals, compared with those of low-income [35]. If true, this phenomenon may be explained by the following: healthy food may still be too expensive for the lowest SES group, even at its most affordable, as the lowest observed healthy-to-unhealthy price ratio for any individual was 1.55. Given that low-income families need to devote approximately half of their food budget to meet the dietary guidelines for fruit and vegetable intake [74], the relative price of healthy food may have to be much lower, and the least healthy food much higher than what was observed in this study. It is also possible that those with the lowest levels of income/wealth may be receiving food assistance (food stamps/supplementary nutrition assistance), which may slightly reduce their price sensitivity. 

Sensitivity to unmeasured confounding—using instrumental variable analyses—confirmed odd ratios found in the main analyses. The IV analysis adds some face validity to the standard regression results; however, as with all models, IV methods have assumptions [66], some of which are not verifiable—notably the third assumption prohibiting any association between the IV and unmeasured cofounders [65]. Confidence intervals from the IV analysis were wider than those obtained from the primary analysis. Wider confidence intervals are to be expected due to the two stage structure of the IV models, which only uses part of the variance in the second stage, and because the prediction of the price ratio by the IV was imperfect [66].

### Limitations

While the validity of the FFQ used in this study has been documented [38,40] the limitations to FFQ are well-known [75]. The FFQ contained an a priori food list that may miss foods consumed by a diverse population; however, a strength of the MESA FFQ was that it included many foods that reflect the diversity of the multi-ethnic population. By defining a high-quality diet relative to the population (top quintile), we attempted to address some of its limitations regarding underreporting. Selection bias may have been introduced, by limiting analyses to individuals living within three miles of a supermarket. However, an examination of the characteristics of those included and excluded found few differences with the exception of region and race/ethnicity, which was due to the excluded being largely concentrated in a single recruitment site where supermarket data were unavailable. 

It is also unknown whether individuals shopped for food at the stores within three miles of their residences; however, it is in-line with prior research examining the average distance individuals travel to their primary supermarkets [59,60,61,62]. We did not have information on the work location of individuals or data on their shopping habits—such as using a supermarket on their route home from work, which may not be located proximal to either their work or home.

Other measurements of food price may have been considered for this analysis, including the price per unit of weight, or price per calorie [6,76]; however, we chose the price per serving, as it is a unit of analysis that can be compared across all product types, may be most meaningful to consumers, and has been used previously in similar research [7,8,9]. 

There are limitations to the products included in the price measures. Since data for fresh fruits and vegetables were not available, refrigerated products were selected to roughly approximate costs of fresh fruit and vegetable spoilage and storage/distribution, and proxy fresh produce. Healthy food was represented by dairy (refrigerated milk, yogurt, cottage cheese), fruits and vegetables (fresh orange juice and frozen vegetables). It is unknown how the results would have changed if we had access to data on fresh fruits and vegetables. However, it is plausible that our results would be roughly similar if fresh fruit and vegetables were used because (1) the price of fresh oranges correlates well with the price of refrigerated fresh orange juice [55], and (2) the price of frozen vegetables was only one component of our healthy food measurement. Nevertheless—among frozen vegetables commonly purchased—one report found average frozen vegetable prices were similar or somewhat cheaper than fresh. For example, prices of frozen vegetable per edible cup were nearly identical (broccoli), frozen much cheaper (spinach, corn), frozen slightly cheaper (cauliflower), and frozen slightly more expensive (carrots) [9]. Furthermore, data on other healthy foods, such as whole grains and legumes, were not available. Data were limited to branded products in order to compare the same products across the country. Finally, this study was cross-sectional, and thus, causality between food prices and diet cannot be inferred. While the evidence presented in this paper suggests an association, more evidence—specifically from prospective studies—is needed to fully understand how food prices influence purchasing decisions and subsequent diet quality.

## 5. Conclusions

With a large proportion of the U.S. population failing to achieve a healthy diet, it is important to understand the root causes. This study suggests that the larger differences in price between healthy and unhealthy food may play a role. If this is true, interventions may be considered to improve the affordability of healthy foods relative to unhealthy alternatives. 

## Figures and Tables

**Table 1:** Characteristics of the individuals included in the analysis by tertiles of healthy-to-unhealthy food price ratio (*n* = 2642).

Characteristic	All Participants		Lowest Ratio, Smallest Differential		Middle Ratio		Highest Ratio, Largest Differential	
			(1.55–1.88)		(1.88–2.01)		(2.01–2.39)	
	*n* or Mean	Col % or SD	*n* or Mean	Col % or SD	*n* or Mean	Col % or SD	*n* or Mean	Col % or SD
**Number of participants** (*n*)	2765		938		903		924	
**MESA recruitment site** (*n*, %) **^a^**								
Forsyth County, NC	539	19.5%	239	25.5%	299	33.1%	1	0.1%
New York, NY	538	19.5%	262	27.9%	226	25.0%	50	5.4%
Baltimore, MD	464	16.8%	339	36.1%	122	13.5%	3	0.3%
St. Paul, MN	8	0.3%	6	0.6%	0	0.0%	2	0.2%
Chicago, IL	651	23.5%	89	9.5%	221	24.5%	341	36.9%
Los Angeles, CA	565	20.4%	3	0.3%	35	3.9%	527	57.0%
**Region of residence** (*n*, %)								
Northeast	534	19.3%	259	27.6%	226	25.0%	49	5.3%
Midwest	642	23.2%	85	9.1%	220	24.4%	337	36.5%
South	1021	36.9%	593	63.2%	421	46.6%	7	0.8%
West	568	20.5%	1	0.1%	36	4.0%	531	57.5%
**Supermarket density** (3 mile) (mean, SD)	1.19	1.42	1.51	1.88	1.22	1.3	0.84	0.74
**Female** (*n*, %)	1466	53.0%	483	51.5%	502	55.6%	481	52.1%
**Age** (mean, SD)	70.3	9.5	70.6	9.1	70.3	9.3	69.9	9.9
**Race/ethnicity** (*n*, %)								
White	1101	39.8%	422	45.0%	485	53.7%	194	21.0%
Chinese American	359	13.0%	10	1.1%	56	6.2%	293	31.7%
Black	834	30.2%	413	44.0%	231	25.6%	190	20.6%
Hispanic	471	17.0%	93	9.9%	131	14.5%	247	26.7%
**Education** (*n*, %)								
High school diploma or less	777	28.1%	231	24.6%	251	27.8%	295	31.9%
Some college	761	27.5%	259	27.6%	233	25.8%	269	29.1%
Bachelor’s degree or more	1227	44.4%	448	47.8%	419	46.4%	360	39.0%
**Income**								
Per capita household income (in $10k) (mean, SD)	2.6	1.9	2.8	1.8	2.8	1.9	2.3	1.8
Wealth index	2.6	1.2	2.6	1.2	2.6	1.2	2.5	1.2
Income/wealth index	5.1	2.2	5.3	2.1	5.2	2.2	4.9	2.3
**Marital status** (*n*, %)								
Not married or living with partner	1107	40.0%	419	44.7%	364	40.3%	324	35.1%
Married/Living w. partner	1658	60.0%	519	55.3%	539	59.7%	600	64.9%
**Body mass index** (mean, SD)	28.2	5.6	28.9	5.5	28.4	5.9	27.2	5.2
<25 (*n*, %)	855	30.9%	236	25.2%	268	29.7%	351	38.0%
25–29.9 (*n*, %)	1043	37.7%	347	37.0%	341	37.8%	355	38.4%
≥30 (*n*, %)	867	31.4%	355	37.8%	294	32.6%	218	23.6%
**Smoking status** (*n*, %)								
Never smoked	1281	46.3%	366	39.0%	412	45.6%	503	54.4%
Former smoker	1283	46.4%	487	51.9%	433	48.0%	363	39.3%
Current smoker	201	7.3%	85	9.1%	58	6.4%	58	6.3%
**Physical activity, MET min per week** (mean, SD)	2774	3552	3239	4237	2765	3441	2310	2749

**^a^** This is the MESA location of the participant, not necessarily their area of residence.

**Table 2:** Proportion of participants with a high-quality diet by tertile of the healthy-to-unhealthy price ratio and the average serving price of healthy and unhealthy foods (*n* = 2765).

Variable	All Participants			Lowest Ratio (1.55–1.88)			Middle Ratio (1.88–2.01)			Highest Ratio (2.01–2.39)		
**Number of participants** (*n*)	2765			938			903			924		
**Number with high-quality diet** (*n*, %)	545	19.7%		165	17.6%		161	17.8%		219	23.7%	
**Average food prices per serving**												
Healthy food price per serving **^a^** (mean [SD], median)	$0.60	[$0.04]	$0.60	$0.61	[$0.06]	$0.57	$0.59	[$0.03]	$0.58	$0.62	[$0.03]	$0.62
Unhealthy food price per serving **^b^** (mean [SD], median)	$0.31	[$0.03]	$0.30	$0.33	[$0.04]	$0.31	$0.30	[$0.01]	$0.30	$0.29	[$0.01]	$0.29
Ratio of healthy-to-unhealthy (mean [SD], median)	1.97	[0.14]	1.93	1.83	[0.05]	1.85	1.95	[0.05]	1.93	2.14	[0.09]	2.13

**^a^** Healthy foods were represented by frozen vegetables, orange juice, and dairy (milk, yogurt, and cottage cheese); **^b^** Unhealthy foods were represented by soda, salty snacks (chips, pretzels, onion rings), and sweets (chocolate candy and cookies).

**Table 3:** Odds ratios of having a high-quality diet associated with the price ratio, and with the prices of healthy foods and unhealthy foods after sequential adjustment for confounders within the full population (*n* = 2765).

Model Covariates	Exposure of Interest											
	Healthy-To-Unhealthy Ratio				Healthy Food Price				Unhealthy Food Price			
	95% CI				95% CI				95% CI			
	Odds Ratio	Lower	Upper	*p* Value	Odds Ratio	Lower	Upper	*p* Value	Odds Ratio	Lower	Upper	*p* Value
**Full sample** (*n* = 2765)												
**Model 1**: region, age, gender	0.97	0.83	1.13	0.6978	0.98	0.88	1.10	0.7655	1.00	0.91	1.10	0.9639
**Model 2**: Model 1 plus income/wealth, education level, smoking status, and race	0.86	0.73	1.01	0.0571	0.97	0.86	1.09	0.5709	1.03	0.93	1.13	0.6045
**Final Model**: Model 2 plus neighborhood SES **^a^** and neighborhood supermarket density	0.76	0.64	0.91	0.0027	1.04	0.88	1.22	0.6371	1.16	1.02	1.33	0.0267

**^a^** Neighborhood SES was derived from log of the median household income; log of the median value of housing units; the percentage of households receiving interest, dividend, or net rental income; the percentage of adults 25 years of age or older who had completed high school; the percentage of adults 25 years of age or older who had completed college; and the percentage of employed persons 16 years of age or older in executive, managerial, or professional specialty occupations.

**Table 4:** Odds ratios of having a high-quality diet associated with the price ratio, and with the prices of healthy foods and unhealthy foods, stratified by wealth/income and by education (*n* = 2765).

Socioeconomic Status Measure					Exposure of Interest							
	Healthy-To-Unhealthy Ratio				Healthy Food Price				Unhealthy Food Price			
		95% CI				95% CI				95% CI		
	OR	Lower	Upper	*p* Value	OR	Lower	Upper	*p* Value	OR	Lower	Upper	*p* Value
**Wealth/income tertile ^a^**												
Lowest (1–4), *n* = 956	0.79	0.58	1.06	0.1149	0.99	0.76	1.28	0.9200	1.16	0.90	1.49	0.2654
Middle (5–6), *n* = 793	0.61	0.43	0.87	0.0067	1.13	0.81	1.57	0.4746	1.32	1.02	1.70	0.0365
Highest (7–8), *n* = 893	0.87	0.64	1.18	0.3621	1.11	0.83	1.49	0.4846	1.10	0.89	1.36	0.3825
**Education level ^b^**												
HS degree or less, *n* = 777	0.85	0.58	1.24	0.3897	0.79	0.58	1.09	0.1513	0.90	0.65	1.25	0.5354
Some college, *n* = 761	0.81	0.57	1.16	0.2552	1.11	0.78	1.59	0.5586	1.20	0.89	1.62	0.2339
Bachelor’s degree or more, *n* = 1227	0.77	0.59	0.99	0.0412	1.15	0.91	1.47	0.2512	1.18	0.99	1.41	0.0621

**^a^** *p* value for interaction between wealth/income and the price ratio *p* = 0.0012; healthy price *p* = 0.4047; unhealthy price *p* = 0.0053; **^b^** *p* value for interaction between education level and the price ratio *p* = 0.0949; healthy price *p* = 0.4415; unhealthy price *p* = 0.2111.

**Table 5:** Instrumental variable analysis results using toilet paper as the instrument and two-stage residual inclusion models for the healthy-to-unhealthy price ratio, healthy food price, and unhealthy food price (*n* = 2765) **^a^**.

Price Outcome	*n*	*n* Events	Stage 1		Stage 2		
			*t*-Value	F-Statistic	Odds Ratio	Lower CL ^b^	Upper CL ^b^
Healthy-to-unhealthy price ratio	2765	543	-33.36	1113	0.82	0.57	1.19
Healthy food price	2765	543	59.86	3583	1.15	0.91	1.45
Unhealthy food price	2765	543	98.59	9720	1.10	0.93	1.30

**^a^** Covariates in each stage included age, gender, geographic region, wealth/income index, race, smoking status, neighborhood SES, supermarket density, population density, and cost of living index; **^b^** Confidence limits were obtained from a bootstrapped analysis using 10,000 replications.

## Supplementary Materials

The following are available online at www.mdpi.com/1660-4601/14/11/1394/s1. Table S1: Serving sizes and weights for composite price calculations using two different weight calculations; Table S2: Characteristics of included and excluded individuals in the analysis of diet quality; Table S3: Results of sensitivity analyses using equal weights for the price outcomes, using a five-mile radius to capture prices for all individuals, and using a one-mile radius for those living in New York City and a three-mile radius for all others.

## References

[1] Connors M., Bisogni C.A., Sobal J., Devine C.M. (2001). Managing values in personal food systems. Appetite.

[2] Andreyeva T., Long M.W., Brownell K.D. (2010). The impact of food prices on consumption: A systematic review of research on the price elasticity of demand for food. Am. J. Public Health.

[3] Aggarwal A., Rehm C.D., Monsivais P., Drewnowski A. (2016). Importance of taste, nutrition, cost and convenience in relation to diet quality: Evidence of nutrition resilience among U.S. adults using National Health and Nutrition Examination Survey (NHANES) 2007–2010. Prev. Med..

[4] Hausman J.A., Leonard G.K. (2002). The competitive effects of a new product introduction: A case study. J. Ind. Econ..

[5] Drewnowski A. (2010). The cost of U.S. foods as related to their nutritive value. Am. J. Clin. Nutr..

[6] Andrieu E., Darmon N., Drewnowski A. (2006). Low-Cost diets: More energy, fewer nutrients. Eur. J. Clin. Nutr..

[7] Lipsky L.M. (2009). Are energy-dense foods really cheaper? Reexamining the relation between food price and energy density. Am. J. Clin. Nutr..

[8] Reed J., Frazão E., Itskowitz R. (2004). How Much Do Americans Pay for Fruits and Vegetables?.

[9] Stewart H., Hyman J., Buzby J.C., Frazao E., Carlson A. (2011). How Much Do Fruits and Vegetables Cost?.

[10] Kern D.M., Auchincloss A.H., Ballister L., Robinson L.F. (2016). Neighbourhood variation in the price of soda relative to milk and its association with neighbourhood socio-economic status and race. Public Health Nutr..

[11] Rehm C.D., Monsivais P., Drewnowski A. (2011). The quality and monetary value of diets consumed by adults in the United States. Am. J. Clin. Nutr..

[12] Ryden P.J., Hagfors L. (2011). Diet cost, diet quality and socio-economic position: How are they related and what contributes to differences in diet costs?. Public Health Nutr..

[13] Schroder H., Marrugat J., Covas M.I. (2006). High monetary costs of dietary patterns associated with lower body mass index: A population-based study. Int. J. Obes..

[14] Darmon N., Drewnowski A. (2015). Contribution of food prices and diet cost to socioeconomic disparities in diet quality and health: A systematic review and analysis. Nutr. Rev..

[15] Bang J.T., Mitra A., Wunnava P.V. (2015). Financial liberalization and remittances: Recent panel evidence. J. Int. Trade Econ. Dev..

[16] Anekwe T.D., Rahkovsky I. (2014). The association between food prices and the blood glucose level of U.S. adults with type 2 diabetes. Am. J. Public Health.

[17] Powell L.M., Chaloupka F.J. (2009). Food prices and obesity: Evidence and policy implications for taxes and subsidies. Milbank Q..

[18] Guo X., Warden B.A., Paeratakul S., Bray G.A. (2004). Healthy eating index and obesity. Eur. J. Clin. Nutr..

[19] Tande D.L., Magel R., Strand B.N. (2010). Healthy eating index and abdominal obesity. Public Health Nutr..

[20] Chiuve S.E., Fung T.T., Rimm E.B., Hu F.B., McCullough M.L., Wang M., Stampfer M.J., Willett W.C. (2012). Alternative dietary indices both strongly predict risk of chronic disease. J. Nutr..

[21] McCullough M.L., Feskanich D., Rimm E.B., Giovannucci E.L., Ascherio A., Variyam J.N., Spiegelman D., Stampfer M.J., Willett W.C. (2000). Adherence to the dietary guidelines for Americans and risk of major chronic disease in men. Am. J. Clin. Nutr..

[22] Arem H., Reedy J., Sampson J., Jiao L., Hollenbeck A.R., Risch H., Mayne S.T., Stolzenberg-Solomon R.Z. (2013). The healthy eating index 2005 and risk for pancreatic cancer in the NIH-AARP study. J. Natl. Cancer Inst..

[23] Li W.Q., Park Y., McGlynn K.A., Hollenbeck A.R., Taylor P.R., Goldstein A.M., Freedman N.D. (2014). Index-based dietary patterns and risk of incident hepatocellular carcinoma and mortality from chronic liver disease in a prospective study. Hepatology.

[24] Rathod A.D., Bharadwaj A.S., Badheka A.O., Kizilbash M., Afonso L. (2012). Healthy eating index and mortality in a nationally representative elderly cohort. Arch. Intern. Med..

[25] Kappeler R., Eichholzer M., Rohrmann S. (2013). Meat consumption and diet quality and mortality in NHANES III. Eur. J. Clin. Nutr..

[26] Harmon B.E., Boushey C.J., Shvetsov Y.B., Ettienne R., Reedy J., Wilkens L.R., Le Marchand L., Henderson B.E., Kolonel L.N. (2015). Associations of key diet-quality indexes with mortality in the multiethnic cohort: The dietary patterns methods project. Am. J. Clin. Nutr..

[27] Reedy J., Krebs-Smith S.M., Miller P.E., Liese A.D., Kahle L.L., Park Y., Subar A.F. (2014). Higher diet quality is associated with decreased risk of all-cause, cardiovascular disease, and cancer mortality among older adults. J. Nutr..

[28] Volpe R., Okrent A. (2012). Assessing the Healthfulness of Consumers’ Grocery Purchases.

[29] Ball K., Crawford D., Mishra G. (2006). Socio-economic inequalities in women’s fruit and vegetable intakes: A multilevel study of individual, social and environmental mediators. Public Health Nutr..

[30] De Irala-Estevez J., Groth M., Johansson L., Oltersdorf U., Prattala R., Martinez-Gonzalez M.A. (2000). A systematic review of socio-economic differences in food habits in Europe: Consumption of fruit and vegetables. Eur. J. Clin. Nutr..

[31] Dubowitz T., Heron M., Bird C.E., Lurie N., Finch B.K., Basurto-Davila R., Hale L., Escarce J.J. (2008). Neighborhood socioeconomic status and fruit and vegetable intake among whites, blacks, and Mexican Americans in the United States. Am. J. Clin. Nutr..

[32] Monsivais P., Aggarwal A., Drewnowski A. (2012). Are socio-economic disparities in diet quality explained by diet cost?. J. Epidemiol. Community Health.

[33] Konttinen H., Sarlio-Lahteenkorva S., Silventoinen K., Mannisto S., Haukkala A. (2013). Socio-economic disparities in the consumption of vegetables, fruit and energy-dense foods: The role of motive priorities. Public Health Nutr..

[34] Powell L.M., Zhao Z., Wang Y. (2009). Food prices and fruit and vegetable consumption among young American adults. Health Place.

[35] Darmon N., Lacroix A., Muller L., Ruffieux B. (2016). Food price policies may improve diet but increase socioeconomic inequalities in nutrition. World Rev. Nutr. Diet..

[36] Beydoun M.A., Powell L.M., Wang Y. (2008). The association of fast food, fruit and vegetable prices with dietary intakes among U.S. adults: Is there modification by family income?. Soc. Sci. Med..

[37] Bild D.E., Bluemke D.A., Burke G.L., Detrano R., Diez Roux A.V., Folsom A.R., Greenland P., Jacob D.R., Kronmal R., Liu K. (2002). Multi-ethnic study of atherosclerosis: Objectives and design. Am. J. Epidemiol..

[38] Mayer-Davis E.J., Vitolins M.Z., Carmichael S.L., Hemphill S., Tsaroucha G., Rushing J., Levin S. (1999). Validity and reproducibility of a food frequency interview in a multi-cultural epidemiology study. Ann. Epidemiol..

[39] Nettleton J.A., Steffen L.M., Mayer-Davis E.J., Jenny N.S., Jiang R., Herrington D.M., Jacobs D.R. (2006). Dietary patterns are associated with biochemical markers of inflammation and endothelial activation in the Multi-Ethnic Study of Atherosclerosis (MESA). Am. J. Clin. Nutr..

[40] Nettleton J.A., Rock C.L., Wang Y., Jenny N.S., Jacobs D.R. (2009). Associations between dietary macronutrient intake and plasma lipids demonstrate criterion performance of the Multi-Ethnic Study of Atherosclerosis (MESA) food-frequency questionnaire. Br. J. Nutr..

[41] Nettleton J.A., Schulze M.B., Jiang R., Jenny N.S., Burke G.L., Jacobs D.R. (2008). A priori-defined dietary patterns and markers of cardiovascular disease risk in the Multi-Ethnic Study of Atherosclerosis (MESA). Am. J. Clin. Nutr..

[42] Gao S.K., Beresford S.A., Frank L.L., Schreiner P.J., Burke G.L., Fitzpatrick A.L. (2008). Modifications to the healthy eating index and its ability to predict obesity: The multi-ethnic study of atherosclerosis. Am. J. Clin. Nutr..

[43] Abiemo E.E., Alonso A., Nettleton J.A., Steffen L.M., Bertoni A.G., Jain A., Lutsey P.L. (2013). Relationships of the mediterranean dietary pattern with insulin resistance and diabetes incidence in the Multi-Ethnic Study of Atherosclerosis (MESA). Br. J. Nutr..

[44] Nettleton J.A., Steffen L.M., Ni H., Liu K., Jacobs D.R. (2008). Dietary patterns and risk of incident type 2 diabetes in the Multi-Ethnic Study of Atherosclerosis (MESA). Diabetes Care.

[45] Levitan E.B., Ahmed A., Arnett D.K., Polak J.F., Hundley W.G., Bluemke D.A., Heckbert S.R., Jacobs D.R., Nettleton J.A. (2016). Mediterranean diet score and left ventricular structure and function: The multi-ethnic study of atherosclerosis. Am. J. Clin. Nutr..

[46] Kennedy E.T., Ohls J., Carlson S., Fleming K. (1995). The healthy eating index: Design and applications. J. Am. Diet. Assoc..

[47] U.S. Department of Agriculture (2010). Dietary Guidelines for Americans.

[48] Guenther P.M., Casavale K.O., Reedy J., Kirkpatrick S.I., Hiza H.A., Kuczynski K.J., Kahle L.L., Krebs-Smith S.M. (2013). Update of the healthy eating index: HEI-2010. J. Acad. Nutr. Diet..

[49] Guenther P.M., Kirkpatrick S.I., Reedy J., Krebs-Smith S.M., Buckman D.W., Dodd K.W., Casavale K.O., Carroll R.J. (2014). The healthy eating index-2010 is a valid and reliable measure of diet quality according to the 2010 dietary guidelines for Americans. J. Nutr..

[50] NCI The Healthy Eating Index—HEI Tools for Researchers—Healthy Eating Index-2010. National Cancer Institute, Applied Research Program, Risk Factor Monitoring and Methods. http://riskfactor.cancer.gov/tools/hei.

[51] National Cancer Institute Food Frequency Questionnaire at a Glance. https://dietassessmentprimer.cancer.gov/profiles/questionnaire/.

[52] Bronnenberg B.J., Kruger M.W., Mela C.F. (2008). Database paper—The IRI marketing data set. Mark. Sci..

[53] IRI About US. https://www.iriworldwide.com/en-US/company/about-IRI.

[54] IRI Academic Data Set. http://www.iriworldwide.com/solutions/Academic-Data-Set.

[55] Morris A. Impact of Orange Juice Market Dynamics on Fruit Prices. Proceedings of the International Citrus Economics Conference.

[56] United States Food and Drug Administration CFR—Code of Federal Regulations Title 21, 21CFR101.12. http://www.accessdata.fda.gov/scripts/cdrh/cfdocs/cfcfr/cfrsearch.cfm?fr=101.12.

[57] Diez Roux A.V., Stein Merkin S., Arnett D., Chambless L., Massing M., Nieto J., Sorlie P., Szklo M., Tyroler H.A., Watson R.L. (2001). Neighborhood of residence and incidence of coronary heart disease. N. Engl. J. Med..

[58] The Council for Community and Economic Research (C2ER) Cost of Living Index Manual. https://www.coli.org/surveyforms/colimanual.pdf.

[59] The Capitol Forum (2014). Albertsons/Safeway: Significant Overlaps Likely to Lead to Divestitures; FTC Bureau of Competition Chief Could Lead to More Industry-Friendly Review, but State AGs Present Additional Deal Risk. http://thecapitolforum.com/wp-content/uploads/2014/04/ALB-SAFEWAY-2014.03.20.pdf.

[60] Drewnowski A., Aggarwal A., Hurvitz P.M., Monsivais P., Moudon A.V. (2012). Obesity and supermarket access: Proximity or price?. Am. J. Public Health.

[61] Fuller D., Cummins S., Matthews S.A. (2013). Does transportation mode modify associations between distance to food store, fruit and vegetable consumption, and BMI in low-income neighborhoods?. Am. J. Clin. Nutr..

[62] Michimi A., Wimberly M.C. (2010). Associations of supermarket accessibility with obesity and fruit and vegetable consumption in the conterminous United States. Int. J. Health Geogr..

[63] Greenland S. (2000). An introduction to instrumental variables for epidemiologists. Int. J. Epidemiol..

[64] Hernan M.A., Robins J.M. (2006). Instruments for causal inference: An epidemiologist’s dream?. Epidemiology.

[65] Martens E.P., Pestman W.R., de Boer A., Belitser S.V., Klungel O.H. (2006). Instrumental variables: Application and limitations. Epidemiology.

[66] Rassen J.A., Brookhart M.A., Glynn R.J., Mittleman M.A., Schneeweiss S. (2009). Instrumental variables I: Instrumental variables exploit natural variation in nonexperimental data to estimate causal relationships. J. Clin. Epidemiol..

[67] Terza J.V., Basu A., Rathouz P.J. (2008). Two-Stage residual inclusion estimation: Addressing endogeneity in health econometric modeling. J. Health Econ..

[68] Carlson A., Frazão E. (2012). Are Healthy Foods Really More Expensive? It Depends on How You Measure the Price.

[69] Walker R.E., Keane C.R., Burke J.G. (2010). Disparities and access to healthy food in the United States: A review of food deserts literature. Health Place.

[70] Thow A.M., Downs S., Jan S. (2014). A systematic review of the effectiveness of food taxes and subsidies to improve diets: Understanding the recent evidence. Nutr. Rev..

[71] Powell L.M., Chriqui J.F., Khan T., Wada R., Chaloupka F.J. (2013). Assessing the potential effectiveness of food and beverage taxes and subsidies for improving public health: A systematic review of prices, demand and body weight outcomes. Obes. Rev..

[72] Epstein L.H., Jankowiak N., Nederkoorn C., Raynor H.A., French S.A., Finkelstein E. (2012). Experimental research on the relation between food price changes and food-purchasing patterns: A targeted review. Am. J. Clin. Nutr..

[73] O’Hara J., Musicu A. Big Soda vs. Public Health. Center for Science in the Public Interest: How the Industry Opens Its Checkbook to Defeat Health Measures. https://cspinet.org/file/6465/download?token=qLEj8qfC.

[74] Cassady D., Jetter K.M., Culp J. (2007). Is price a barrier to eating more fruits and vegetables for low-income families?. J. Am. Diet. Assoc..

[75] Willett W.C., Hu F.B. (2006). Not the time to abandon the food frequency questionnaire: Point. Cancer Epidemiol. Biomark. Prev..

[76] Drewnowski A. (2009). Obesity, diets, and social inequalities. Nutr. Rev..

