# Supplementary material for: Neighborhood Prices of Healthier and Unhealthier Foods and Associations with Diet Quality: Evidence from the Multi-Ethnic Study of Atherosclerosis
Source: Int J Environ Res Public Health. 2017 Nov 16;14(11):1394. doi: 10.3390/ijerph14111394 (PMC5708033; doi:10.3390/ijerph14111394)
Supplement: Supplementary file 1 [file ijerph-14-01394-s001.pdf]

International Journal of
Environmental Research and Public Health

MDPI

Supplementary Materials: Neighborhood Prices of Healthier and Unhealthier Foods and Associations with Diet Quality: Evidence from the Multi-Ethnic Study of Atherosclerosis

David M. Kern [1], Amy H. Auchnicloss [1,*], Mark F. Stehr [2], Ana V. Diez Roux [3], Latetia V. Moore [4], Genevieve P. Kanter [5] and Lucy F. Robinson [1]

Table S1. Serving sizes and weights for composite price calculations using two different weight calculations—based on national consumption averages and using equal weights within each food class.

Column Heading	Products Included	Serving Size	Servings Per Day	Weight [a]	Weight [b]
Healthier foods					
Fruits and vegetables [c]	Orange juice, frozen vegetables	1 cup	2.63	0.617	0.50
Dairy [d]	1%, 2% and non-fat refrigerated milk; low fat and non-fat yogurt; and 1%, 2%, and 4% milkfat cottage cheese	8 fl oz	1.63	0.383	0.50
Unhealthy foods					
Soda [e]	Sugar sweetened only	8 fl oz	0.73	0.450	0.33
Sweets [f,g]	Chocolate candy, cookies	30 g/40 g	0.62	0.383	0.33
Salty snacks [h]	Chips, pretzels, tortilla chips, etc.	30 g	0.27	0.167	0.33

[a] Weight based on national consumption averages (servings per day column); [b] Equal weight given to all products in their respective class; [c] NHANES 2011–2012 survey found adults 20 years and older consumed 1.64 cups of vegetables per day and 0.99 cups of fruit per day(United States Department of Agriculture and Agricultural Research Service, 2014); [d] NHANES 2011–2012 survey found adults 20 years and older consumed 1.63 cups of dairy per day. (United States Department of Agriculture and Agricultural Research Service, 2014); [e] Adults ≥60 years old consume 68 calories from SSBs on a daily basis.(Kit et al., 2013) A 12 ounce can of Coca-Cola contains 140 calories,(Coca-Cola, 2014) thus 68 calories equates to 5.8 ounces, or 0.73 daily servings; [f] According to a Canadian agriculture report, 2010 cookie sales was 1 million tons in the United States,(Agriculture and Agri-food Canada, 2012) which equates to 0.27 servings per day; [g] According to the latest report from the International Cocoa Organization, in 2008 Americans consumed 5.09 kilograms of chocolate on average, roughly 11.2 pounds, which equates to 0.49 ounces per day, or 0.35 servings.(International Cocoa Organization); [h] A 2004 report from the USDA found that 95.5% of households consumed any chips (potato, corn, etc.) and of those that did eat chips they consumed 7.0 pounds per capita.(Kuchler et al., 2004) After accounting for those that did not buy any chips this becomes 6.7 pounds per capita annually, 0.29 ounces per day, or 0.27 servings; Abbreviations: g = grams, fl oz = fluid ounces.

Table S2. Characteristics of included and excluded individuals in the analysis of diet quality.

Column Heading	**Included Participants**		**Excluded Individuals**	
	N/Mean	Col %/SD	*n*/Mean	%/SD
Number of participants (*N*)	2765		1951	
MESA recruitment site (*n*, %) [a]				
Forsyth County, NC	539	19.5%	274	14.0%
New York, NY	538	19.5%	272	13.9%
Baltimore, MD	464	16.8%	194	9.9%
St. Paul, MN	8	0.3%	763	39.1%
Chicago, IL	651	23.5%	225	11.5%
Los Angeles, CA	565	20.4%	223	11.4%
Region of residence (*n*, %)				
Northeast	534	19.3%	241	13.0%
Midwest	642	23.2%	937	50.5%
South	1021	36.9%	452	24.3%
West	568	20.5%	227	12.2%
Total supermarket density (3 mile) (mean, SD)	1.19	1.42	0.79	1.19
Female (*n*, %)	1466	53.0%	1048	53.7%
Age (mean, SD)	70.3	9.5	69.4	9.5
Race/ethnicity (*n*, %)				
White	1101	39.8%	824	42.2%
Chinese American	359	13.0%	182	9.3%
Black	834	30.2%	417	21.4%
Hispanic	471	17.0%	528	27.1%
Education (*n*, %)				
High school diploma or less	777	28.1%	718	36.8%
Some college	761	27.5%	610	31.3%
Bachelor's degree or more	1227	44.4%	615	31.5%
Per capita household income (in $10k)	2.6	1.9	2.3	1.7
Wealth index	2.6	1.2	2.5	1.2
Income/wealth index	5.1	2.2	4.8	2.2
Marital status (*n*, %)				
Not married or living with partner	1107	40.0%	825	42.3%
Married/Living with partner	1658	60.0%	1126	57.7%
BMI (mean, SD)	28.2	5.6	28.9	5.7
<25 (*n*, %)	855	30.9%	512	26.2%
25–29.9 (*n*, %)	1043	37.7%	710	36.4%
≥30 (*n*, %)	867	31.4%	729	37.4%
Smoking status (*n*, %)				
Never smoked	1281	46.3%	841	43.1%
Former smoker	1283	46.4%	938	48.1%
Current smoker	201	7.3%	172	8.8%
Physical activity, MET min per week (mean, SD)	2773.7	3552.0	2548.0	3366.9

[a] This is the MESA location of the participant, not necessarily their area of residence.

International Journal of
Environmental Research and Public Health

MDPI

Table S3. Results of sensitivity analyses using equal weights for the price outcomes, using a five-mile radius to capture prices for all individuals, and using a one-mile radius for those living in New York City and a three-mile radius for all others.

Column Heading	Exposure of Interest											
	Healthy-To-Unhealthy Ratio				Healthy Food Price				Unhealthy Food Price			
		95% CI				95% CI				95% CI		
	Odds Ratio	Lower	Upper	*p* Value	Odds Ratio	Lower	Upper	*p* Value	Odds Ratio	Lower	Upper	*p* Value
**Using equal weights for food prices** [a]												
Model 1: region, age, gender	0.92	0.76	1.11	0.3905	0.96	0.86	1.09	0.552	1.00	0.92	1.10	0.9625
Model 2: Model 1 plus income/wealth, education level, smoking status, and race	0.84	0.69	1.03	0.0869	0.97	0.86	1.10	0.6684	1.02	0.93	1.12	0.6297
Final Model: Model 2 plus neighborhood SES and neighborhood supermarket density	0.72	0.57	0.91	0.0051	1.05	0.90	1.23	0.5163	1.16	1.01	1.32	0.0314
**Using a 5-mile radius for food prices** [b]												
Model 1: region, age, gender	1.01	0.84	1.21	0.9487	0.97	0.85	1.10	0.6063	0.98	0.88	1.09	0.6974
Model 2: Model 1 plus income/wealth, education level, smoking status, and race	0.83	0.68	1.01	0.0562	0.95	0.83	1.07	0.3860	1.01	0.91	1.13	0.8417
Final Model: Model 2 plus neighborhood SES and neighborhood supermarket density	0.78	0.63	0.96	0.0216	0.93	0.79	1.10	0.4071	1.05	0.91	1.21	0.5231
**Using a 1-mile radius for those in NYC** [c]												
Model 1: region, age, gender	0.98	0.85	1.14	0.8098	1.01	0.88	1.16	0.8980	1.03	0.91	1.15	0.6798
Model 2: Model 1 plus income/wealth, education level, smoking status, and race	0.89	0.76	1.04	0.1447	0.98	0.85	1.13	0.7611	1.06	0.93	1.19	0.3815
Final Model: Model 2 plus neighborhood SES and neighborhood supermarket density	0.86	0.73	1.01	0.0608	0.97	0.83	1.13	0.6957	1.07	0.94	1.22	0.2792

[a] Each of the two healthy food products (fruits & vegetables, dairy) received a weight of 0.5, while each of the three unhealthy products (soda, chocolate candy & sweets, salty snacks) received a weight of 0.33. The radius for capturing prices remained at three miles for this sensitivity analysis; [b] All IRI supermarkets within five miles of a participant's place of residence at exam 5 were included.

© 2017 by the authors; licensee MDPI, Basel, Switzerland. This article is an open access article distributed under the terms and conditions of the Creative Commons by Attribution (CC-BY) license (http://creativecommons.org/licenses/by/4.0/).
